# New World’s old disease: cardiac hydatid disease and surgical principles

**DOI:** 10.5830/CVJA-2017-006

**Published:** 2017

**Authors:** Omer Tanyeli, Yuksel Dereli, Ilker Mercan, Niyazi Gormus, Tahir Yuksek

**Affiliations:** Department of Cardiovascular Surgery, Meram Medicine Faculty, Necmettin Erbakan University, Konya, Turkey; Department of Cardiovascular Surgery, Meram Medicine Faculty, Necmettin Erbakan University, Konya, Turkey; Department of Cardiovascular Surgery, Meram Medicine Faculty, Necmettin Erbakan University, Konya, Turkey; Department of Cardiovascular Surgery, Meram Medicine Faculty, Necmettin Erbakan University, Konya, Turkey; Department of Cardiovascular Surgery, Meram Medicine Faculty, Necmettin Erbakan University, Konya, Turkey

**Keywords:** hydatid cyst, cardiac hydatid cyst, Echinococcus granulosus

## Abstract

**Background:**

Hydatid cyst is a parasitic disease caused by infection with the Echinococcus granulosus tapeworm larva. It is a major public health problem in endemic regions. Cardiac involvement of the disease is rare.

**Methods:**

Between 1985 and 2015, 12 patients were admitted to our clinic with a possible diagnosis of cardiac hydatid disease. Of these patients, six (50%) were male and six (50%) were female. Mean age of the patients was 42.6 years.

**Results:**

The most common location of cardiac hydatid disease was left sided (six patients, 50%). Five (41.7%) patients had cysts located in the right heart, whereas one (8.3%) had a cyst in the interventricular septum. Eleven (91.7%) of the patients were operated on via median sternotomy and the remaining one was operated on via a left anterolateral thoracotomy. Ten (83.3%) of the patients were operated on using cardiopulmonary bypass under moderate hypothermia, whereas the remaining two (16.7%) had off-pump surgery. There was no surgical mortality in our series. All patients were discharged with medical therapy (mebendazole or albendazole) for the duration of six months. No recurrences were observed in their follow ups.

**Conclusion:**

Although cardiac hydatid disease is rare, its prevalence seems to have increased in the last decade. Any patient with suspected cardiac symptoms suggesting mass lesions should be considered for a differential diagnosis of cardiac hydatid disease, especially in developing countries. Definitive treatment is removal of the cyst, combined with medical therapy.

## Introduction

Hydatid cyst (HC) is a parasitic disease caused by infection with the Echinococcus granulosus tapeworm larva. It is a major public health problem in endemic regions such as Asia, the Middle East, the Mediterranean region, South America, New Zealand and Australia.^1^

Hydatid disease may be seen in a variety of systems in the human body, most commonly in the liver (70%) and pulmonary region (20%). Cardiac involvement is very rare and comprises about 0.5 to 2% of all cases.^2^ Although there are some reports of different locations, and isolated surgical experiences are reported in the literature, large series are limited. In this article, we report our experience in surgical treatment of cardiac hydatid disease (CHD) with specific surgical steps, and we review the literature, which interestingly, shows an increase in reports of CHD over the last two decades.

## Methods

In the 30 years between 1985 and 2015, 12 patients were admitted to our clinic, either from the Departments of Cardiology or Emergency, with a possible diagnosis of cystic cardiac masses, which were highly suspicious for CHD. Of these patients, six (50%) were male and the remaining six (50%) were female. Mean age of the patients was 42.6 years (ranging from 12–65 years). All patients came from areas where the disease is endemic.

The most common presenting symptom was dyspnoea, palpitations and chest pain resembling coronary artery disease. Among these patients, one had symptoms of pulmonary emboli and one was previously operated on because of HC of the left lung, and recurrent CHD was diagnosed five years after the first operation. She also had HCs on the right lung, liver and spleen. All the other CHDs were diagnosed incidentally. Table 1 demonstrates the clinical and demographic features of the patients.

**Table 1:** Demographic data of the patients operated on due to cardiac hydatid disease (n = 12)

*Age (years), mean (range)*	*42.6 (12–65)*
Gender, n (%)	
Male	6 (50)
Female	6 (50)
Location of hydatid cyst, n (%)	
Right sided	5 (41.7)
Right atrium	2 (16.7)
Right ventricle	2 (16.7)
RVOT	1 (8.3)
Left sided	6 (50)
Left ventricle	5 (41.7)
Left atrium	1 (8.3)
Interventricular septum	1 (8.3)
Surgical procedure: cystectomy and capitonnage, n (%)	
Median sternotomy with CPB	10 (83.4)
Median sternotomy without CPB	1 (8.3)
Left AL thoracotomy without CPB	1 (8.3)

n, number; RVOT, right ventricular outflow tract; AL, anterolateral; CPB, cardiopulmonary bypass.

Routine tests comprising full blood count, and biochemistry and serological tests, including indirect haemagglutination (IHA) and/or enzyme-linked immunosorbent assay (ELISA) were the preferred diagnostic tools. Echocardiography was preferred to define the mass lesions with their haemodynamically active adjacent structures. Radiological tests, including plain chest X-ray, were done on all patients, and computerised tomography (CT) and/or magnetic resonance imaging (MRI) studies were performed in order to exactly define the lesion, such as the nature of the cystic lesion, location, dimensions and the relationship of the mass with the surrounding tissue or any presence of HC in the lungs. Routine abdominal ultrasonography was performed in order to exclude concomitant HC in the abdomen.

After clearly defining the lesion, the patients were given information on the disease and written informed consents were received before the operation. All patients, except one who did not give consent for surgery (not included in this series), were operated on for cystectomy of CHD.

## Results

The most common location of CHD was left sided (six patients, 50%). Five (41.7%) patients had CHD located in the right heart, whereas one (8.3%) had CHD in the interventricular septum. Eleven (91.7%) of the patients were operated on through median sternotomy and the remaining one was operated on via a left anterolateral thoracotomy. Ten (83.3%) patients were operated on using cardiopulmonary bypass (CPB) under moderate hypothermia, whereas the remaining two (16.7%) had surgery without CPB.

During surgery, as previously described, right-sided cardiac hydatid cysts deserve special attention, Our technique is that, while performing the cannulation, initially a single cannula is inserted into the superior vena cava, and after clamping the pulmonary artery with the aorta, the inferior vena cava cannula is inserted in order to avoid iatrogenic HC embolisation.^3^ There are no special precautions regarding left-sided CHCs in terms of cannulation.

**Fig. 1.**
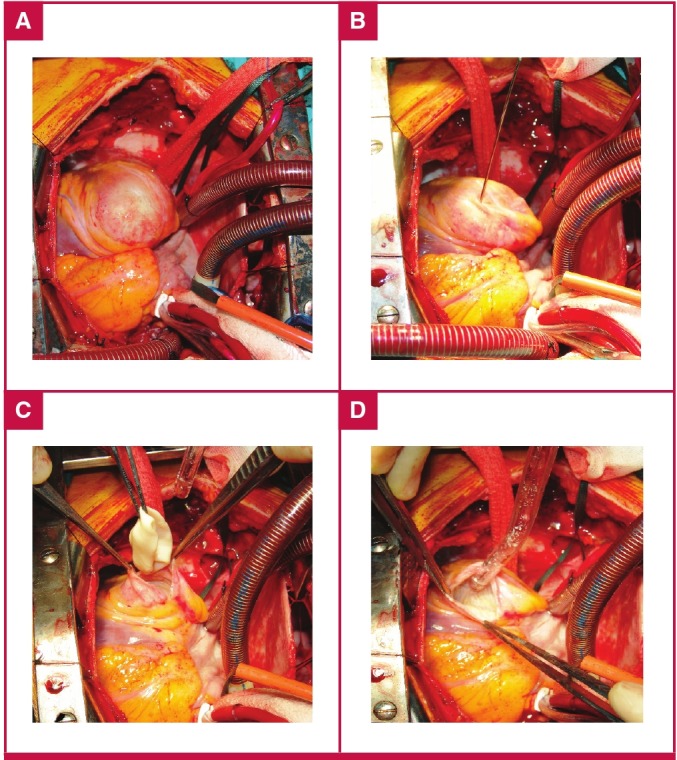
Surgical procedures of cardiac hydatic cystectomy. A. Cardiac hydatid cyst located on the posterior left ventricular wall. B. An aspiration needle inserted into the cyst and 10% hypertonic saline injected into the cystic cavity for sterilisation. C. Removal of the germinative membrane. D. Aspiration of the contents of the cystic cavity.

**Fig. 2.**
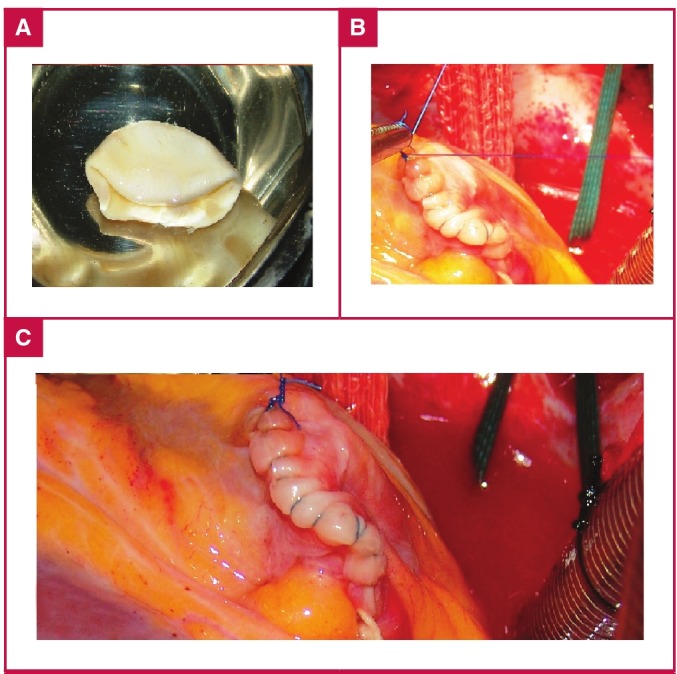
A. Intact germinative membrane. B, C. Closure of the cystic cavity.

After cannulation, the surgical procedure was standard. First the cyst was punctured with a wide aspiration needle connected to the suction device, and after aspiration, without removing the needle, 10% hypertonic saline was injected into the cystic cavity for sterilisation (Fig. 1). The endocyst and the remaining daughter cysts were then removed after gently opening the cystic cavity. Finally, the residual cavity was closed either with continuous or multiple interrupted prolene sutures (Fig. 2).

**Fig. 3.**
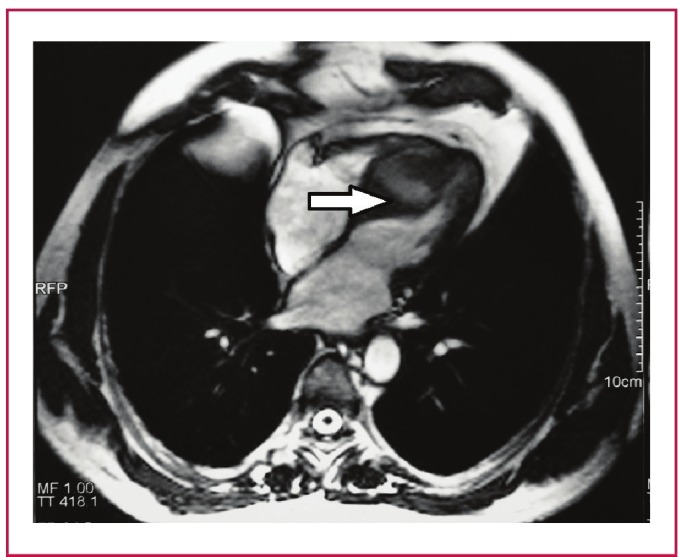
Magnetic resonance image showing the cystic mass in the interventricular septum (white arrow).

In our series, one of the patients, a 58-year-old male, had CHD in the interventricular septum (Fig. 3). The patient had interventricular cystectomy and capitonnage surgery using standard CPB techniques under moderate hypothermia. The patient also had a bypass to the second branch of the circumflex (OM2) artery using a radial artery graft. All the resected material was sent to the Pathology Department and reports indicated either intact or degenerated HC.

There was no surgical mortality in our series. Our patients had neither cardiac rhythm disturbances nor positive inotropic support postoperatively. Mean intensive care unit stay was two days (range between one and three days) after the operation, and seven days (ranging between five and nine days) to discharge from hospital. All patients were followed up with echocardiography. In the first week, the results showed no worsening of left ventricular ejection fraction, compared with pre-operative results.

**Fig. 4.**
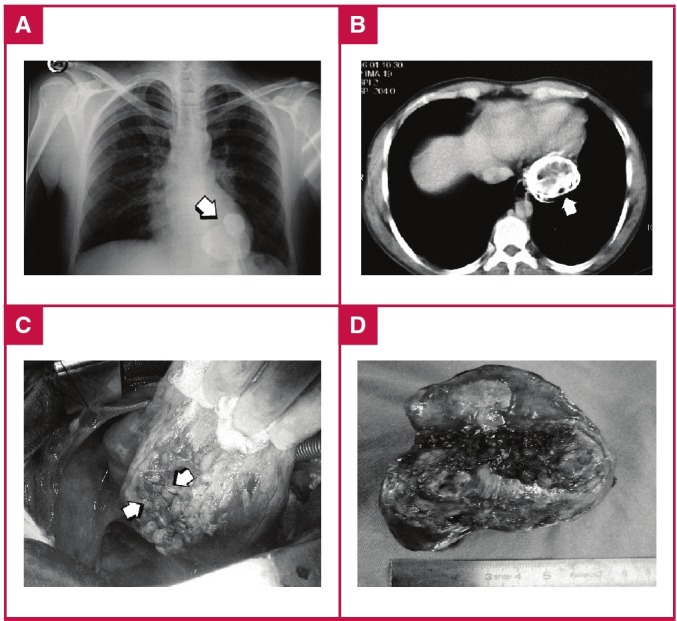
A. Plain chest X-ray showing the calcified outer layer of the cardiac hydatid cyst. B. Eggshell appearance of the cardiac hydatid cyst located on the posterior left ventricular wall on computerised tomography. C, D. Surgically closed defect after removal of the highly calcified hydatid cyst.

All patients were discharged with either mebendazole (in six cases) or albendazole (400 mg twice a day) treatment for six months and all patients, except one, who was operated on one year ago, were followed up for a mean period of 5 ± 2 years. None of the patients had recurrence of CHD. One of our patients who had CHD in the left ventricular posterior wall (Fig. 4) had re-operation two years after cystectomy because of severe mitral insufficiency and the mitral valve was repaired using an annuloplasty ring.

## Discussion

Hydatid cyst is an important parasitic infection caused by the larvae of the Echinococcus granulosus tapeworm. Some carnivores (most often dogs) are the definitive hosts. Although it is believed not to be a common health problem in developed countries, the most common presentation of the disease is in endemic areas such as Asia, the Middle East, the Mediterranean region, South America, New Zealand and Australia.^1^ It may be seen in any organ or tissue in humans, but is most commonly seen in the liver and lung. Cardiac involvement is rare and comprises about 0.5 to 2% of all cases.^2^

The involvement of CHD may be either primary or secondary. Primary involvement of the heart usually occurs via the coronary circulation; the intestinal lymphatics, thoracic duct, vena cavae and patent foramen ovale may be other pathways.^4^ Secondary involvement occurs from dissemination of the cyst from adjacent organs, including the lungs, mediastinal structures or liver through the diaphragm.^5^ The most common involvement of CHD includes the myocardium, mostly in the left ventricle (50–70%), followed by the atria and free wall of the right ventricle (30%), the pericardium (15–25%) and the interventricular septum (5–15%).^6^ In our series, the most common location of the CHD was left sided (six patients, 50%). Five (41.7%) patients had CHD in the right heart, whereas one patient (8.3%) had one in the interventricular septum.

Until the late phase of the disease, patients usually do not seek medical help, probably because it remains asymptomatic for a long period of time. Presenting symptoms of CHD are variable depending on the size, number and location of the cysts.^7^ As the cysts grow and reach reasonable sizes, patients may present with chest pain, palpitations and dyspnoea. Only 10% of patients, particularly those with large HCs have clinical manifestations. Precordial pain is the most common symptom and is most often vague and does not resemble angina pectoris.^8^

When the cyst is located near the valvular apparatus, it may stimulate valvular stenosis or cause valvular regurgitation.^9^ In our series, one patient had severe mitral regurgitation after resection of the HC located over the posterior wall of the left ventricle, which may have been the cause for further mitral valve repair. Although CHD may mimic any valvular pathology, or pericardial or coronary artery disease of the heart, there are no specific symptoms regarding the diagnosis of CHD.

Routine laboratory tests are not specific and may reveal both normal and abnormal results. Blood count may show eosinophilia, but it may also be completely normal. Serological tests such as IHA and ELISA can assist in the diagnosis of HC infection, but since they have a sensitivity of only 80%, false negative results should be considered.^10^

Usually the diagnosis starts with clinical suspicion of the disease. Plain chest X-rays may give negative results in the early phase of the disease. If the HC has a calcified outer layer or has led to an increase in cardiothoracic index, or caused a deformation over the borders of the heart, the X-ray may provide valuable data, but accurate diagnosis is made by echocardiography, CT or MRI studies. Sometimes the cyst may be found incidentally from non-specific radiological or echocardiographic evaluations. In detecting CHD, transthoracic echocardiography should be the first choice, since it is non-invasive with a high sensitivity to demonstrate the mass.

CT or MRI should also be used in order to demonstrate the extent of the cyst and anatomical relationships prior to surgery. CT is superior for observing intracystic gas, minute calcifications and in anatomical mapping.11 Cysts may be identified as uni- or multilocular. Pathognomonic findings are the presence of a single cyst with a wall, daughter cysts surrounded by a capsule with peripheral calcifications, and membrane detachment.^12^

MRI is the most reliable diagnostic modality for CHD; it depicts the exact anatomical location, and the nature of internal and external structures. A typical finding on T2-weighted images is a hypo-intense peripheral ring, representing the pericyst. More specific signs include calcification of the cyst wall, presence of daughter cysts, and membrane detachment.

**Fig. 5.**
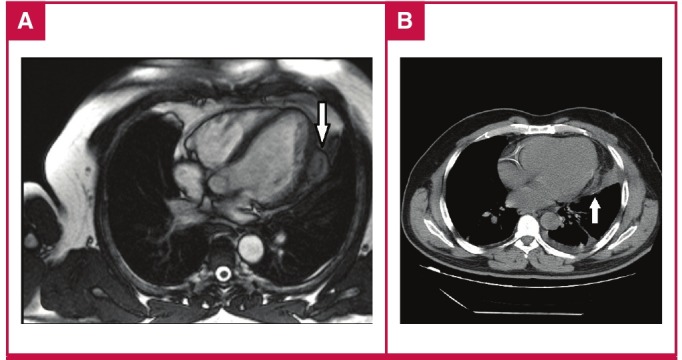
Magnetic resonance (A) and computerised tomography (B) images of the cardiac hydatid cyst located on the left ventricular free wall.

CT best shows wall calcification, whereas MRI depicts the exact anatomical location.^12^ In our clinical practice, we use either CT or MRI prior to surgery to devise a surgical plan and decide accordingly whether to carry out on- or off-pump surgery (Fig. 5).

Estimates on the average increase in cyst diameter vary from about one to 1.5 cm per year.^13^ The disease may be silent for years or cause fatal complications, such as rupture of the HC, resulting in anaphylaxis. Whatever the location, surgical removal of the cyst is the definitive treatment for potentially lifethreatening complications, such as rupture, cardiac tamponade or pulmonary/systemic embolisation.

Most surgeons prefer median sternotomy but on selected and well-defined lesions, left anterolateral thoracotomy may also be used. We performed one operation through left anterolateral thoracotomy in our series. In patients with superficially localised or pericardial CHD, the off-pump technique can be used, as in two of our patients. Using the CPB technique may be mandatory or sometimes beneficial; cross-clamping of the aorta and pulmonary artery may prevent dissemination of the parasite to the systemic or pulmonary circulation, thereby preventing possible pulmonary emboli. After cannulation, our surgical approach is to puncture and aspirate the cyst contents, sterilise with 10% saline solution and close the cavity with purse-string sutures.

After surgery, close follow up of the patients is important to detect any recurrence or dissemination to other organs. Despite successful surgery, supplemental medical therapy should be administered in case of possible cyst rupture and dissemination of daughter cysts during the operation and to prevent recurrence of the cysts.^14^ We recommend albendazole 400 mg twice a day for a period of six months.

**Fig. 6.**
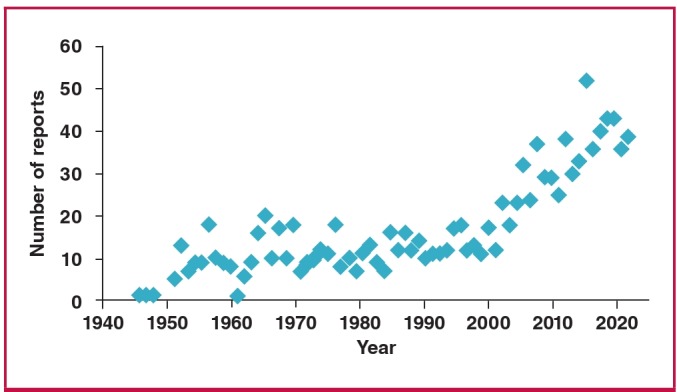
Number of cardiac hydatid cyst reports on PubMed data search.

We searched the literature in the PubMed database using the words ‘cardiac hydatid cyst’ and the number of reports is shown in Fig. 6. As can be seen, the number of reports has increased dramatically over the last decade or two. This may be explained by more accurate diagnosis using either echocardiographic or radiological (CT or MRI) studies, and the increased numbers of open-heart surgery cases since the late 1950s. However, we should keep in mind that, as people travel more and immigrants disperse all over the world, an endemic disease will not remain endemic. Therefore a disease that we thought belonged to the Old World will also be seen in the New World.

## Conclusion

Although CHD is an extremely rare disease, its prevalence seems to have increased in the last decade. Any patient with suspected cardiac symptoms suggestive of mass lesions should be considered for a differential diagnosis of CHD, especially in developing countries. Definitive treatment is removal of the cyst combined with medical therapy. Surgery performed by experienced practitioners provides excellent results when combined with postoperative medical therapy. We will probably see more cases, not only in endemic regions, but also in developed countries in the near future due to the migration of populations.
